# Temporality in Co-generative Processes: Reframing Time in Territorial Complexity

**DOI:** 10.1007/s11213-023-09634-2

**Published:** 2023-02-10

**Authors:** James Karlsen, Clare Hildebrandt

**Affiliations:** Department of Working Life and Innovation, School of Business and Law, University of Agder, Grimstad, Norway

**Keywords:** Time, Temporality, Process time, Linear time, Action research for territorial development, Co-generation of knowledge, Co-generative event

## Abstract

Action research provides fertile grounds for co-generation of knowledge in complex contexts and to be present in the becoming of the process. Time and temporality warrant explanation and distinction, and in retrospective a process can be described with temporal phases, such as planning, observation, action, reflection, evaluation, and modification. Such a description may appear rational, sequential, and linear. However, an action research process is not that! This paper explores the various positionalities of the action researcher, as an insider in a process of becoming, showing how time and temporality can be made explicit in the evolution of an action research process. Our contributions to the action research literature concerning co-generation of knowledge when addressing territorial complexity are: (1) an explicit awareness of temporality provides the opportunity for research on evolvement of processes from the inside, (2) presence in the becoming of a process means there is a unique possibility for reflection and iteration, (3) research in the present tense allows for insight into unexpected developments that create the foundation for future action, as an alternative to retrospective process evaluation, and (4) modelling the process creates a narrative which tells the story of evolution of the process over time.

## Introduction

This article introduces the perspectives of time and temporality in the ongoing processes of territorial knowledge co-generation. In addition to what is known as timely action and actionable knowledge in Action Research protocols, time and temporality have substantial implications for actors engaged in territorial development processes. In our discussion, time is understood as a unit of measure that sets boundaries for knowledge, whereas temporality represents expansion of time from a known past into a yet unknown future. We assert that temporal knowing (knowledge in action across time/place) and paying attention to how we act upon the time-specific knowledge of other disciplines and contexts determine the quality of our collaborations in action and transformative outcomes of actionable knowledge. Temporal knowing and time-specific knowledge are essential in co-generative events and processes. How time-specific knowledge is perceived and acted upon impacts how co-generative knowledge is shaped and experienced in the territorial development processes. By making temporal knowing explicit, it is possible to study and contribute to a process of becoming via the lens of co-generative events. In doing so, action researchers can make openings for new perspectives not only on future potentialities, but also reinterpretations of the past.

In this article, we distinguish between two approaches to time and temporality. The first is the classical, realist approach to time, understood as a natural and objective unit that can be measured and used to organize activities (Ancona et al. 2001). In this approach temporality can be ordered sequentially along a timeline, as linear time with an orientation with the past, the present and the future. They are clearly distinguished, the past as something that has happened, and the future as something that can be desired and constructed (Hernes 2014; Hussenot et al. 2020). Linear time is objective, structured, boundaried, sequenced and ordered. The past does not precede future, it is stable and unchanging. The second approach is founded on the ontological assumption that the world consists of processes, a flow of time, implying that the future is emerging and that there can be a multiplicity of possibilities for future developments (Hernes 2014; Hernes and Schultz 2013; Hussenot and Missonier 2015). This approach uses the orientation of past-time, present-time and future-time. However, the distinction is that these times (past, present, and future) are connected and integrated with one another. In this perception of time, past-time is not separated from the present, and future-time is not dependent on a single understanding of the present, but possibly multiple understandings. The past and the future are not stable entities but can be revised in any present. More conceptual elaborations will follow in the theoretical discussion.

In territorial development processes, time is a moderating factor for actionable knowledge and timely action. To engage in co-generative processes, we advocate for territorial actors (inclusive of action researchers) to have a process awareness that integrates temporal dimensions. Most action researchers acknowledge that change and co-generation of knowledge are gradual processes that take time, but time has become a take-for-granted assumption in research. In this article, we reframe time as a knowledge continuum in its temporal (past-present-future) knowing. We understand and interpret the past with our current knowledge in the present. This implies that the past might be interpreted more nuanced or differently than before with new knowledge. There are many examples of pasts that have been rewritten because of new knowledge. This does not change what happened, but our interpretation of what and why it happened changes. The change of the past happens in the present, which can be understood as an actual moment or event. In process terms, events are temporal experiences (Hernes 2014). The notion of an event is understood as a fact, a moment in which an activity is concretized and made tangible (Hussenot and Missonier 2015). An event can be a meeting, a seminar, a conference etc., and can occur in a short time or stretch out over time. In the actual event of living present, the past and the anticipated future is connected by the actors (Hernes 2014; Hussenot and Missonier 2015). In the living present, interpretations of the past (retrospective) and the future (prospective) can be reinterpreted. This puts the actual event in the foreground, while arguing for the relevance of the past and the future in the experience of the present (Hussenot and Missonier 2015). In this article, we argue as time is passing, that our past understanding can change with present knowledge. This has also implications for our interpretation of the future. Also, the future is interpreted with our current knowledge and as we reinterpret the past, the desired future might be changed. Knowledge of the past is also influencing the construction of the future and future knowing.

## Research Overview

Action research is a family of approaches within a variety of settings and with great diversity of applications (Reason and Bradbury 2008). Our study combines organizational development consulting with action research for territorial development (ARTD) (Karlsen and Larrea 2014) and action research for transformation (Bradbury et al. 2019). Action research is one of OD’s core origins and one of its distinctive features (Coghlan and Shani 2013, p. 524). It is a collaborative, interventionist form of research with roots back to Kurt Lewin’s focus on practice (Coghlan and Shani 2013) and Chris Argyris and Donald Schön’s work of reflective practice (Argyris and Schön 1974, 1996; Schön 1983). One cornerstone of AR is the integration of participants as colearners (Elden and Levin 1991; Greenwood and Levin 2007) that produces better learning and more valid research data (Coghlan and Shani 2013).

The theoretical contribution is founded on the cyclical orientation (process approach) of temporality and the notion of ‘event’ presented in the introduction, as applied in the action research approach for ‘co-generation of knowledge’ (Elden and Levin 1991; Greenwood and Levin 2007; Karlsen and Larrea 2014; Klev and Levin 2012). Knowledge co-generation is a learning process between academics (researchers) and non-academics (practitioners), with the aim of constructing new knowledge that can solve a challenge defined by the actors participating in the process, and which are organized and facilitated by action researchers (Greenwood and Levin 2007; Karlsen and Larrea 2014). Co-creation is a concept that has similarities with co-generation and has become quite much used by academics and policy makers in Norway since 2014 (Røiseland and Lo 2019). In Norwegian co-creation is translated as ‘samskaping’. However, long before ‘samskaping’ became popular, Klev and Levin (2002) used the concept ‘samskapt læring’, which they in their English version of the book has translated to co-generation of knowledge. Action researchers Skipper and Pepler (2020) use co-creation with the same meaning as we uses co-generation. (Klev and Levin 2012). In the following we will use co-generation and co-creation synonymously in ARTD contexts.

### Territorial Complexity

The challenge we explore in this article is territorial complexity. To start, territorial challenges bring together actors in a physical location who become interdependent with one another in dialogue and action (Karlsen and Larrea 2014). Territorial challenges are often difficult due to differing aims and varied interests across the actors, and divergent interpretations of the challenges as viewed by the different territorial actors can further complexify the work (Karlsen 2010). Context matters for knowledge co-generation processes (Karlsen and Larrea 2017), and working with territorial complexity implies collaboration and co-generation of knowledge for shared solutions. There are no easy solutions to territorial complexity, and one actor alone could not solve for the territorial challenge. In this study, we apply action research for territorial development coined by Karlsen and Larrea (2014), which is a modification of the co-generative action research model (Greenwood and Levin 2007; Klev and Levin 2012) adapted to territorial challenge as the context.

### About *Venneslabrua*^1^

To give context to territorial complexity, we utilize a multi-year research and development (R&D) initiative in the Agder Region of Norway. The case is given the name, Project Venneslabrua, and launched in 2019 as a response to a call for holistic and long-term efforts towards betterment of social welfare. The primary goal is to support young adults through vocational education in order to enable their active participation in the workforce. The project period is five years (2019–2024) and based on collaboration with Vennesla videregående skole (Vennesla vgs), which is one of the 17 colleges in Agder. In this supportive employment model, young adults between the ages of 15 and 24 who live in Vennesla Municipality or attend upper secondary school or high school in Vennesla, can benefit from timely follow-up. Project Venneslabrua is formally sponsored by Agder Country, with participation from NAV (Norwegian Labour and Welfare Administration), Vennesla Municipality, and University of Agder. This program is staffed with five members with qualifications to work with those at risk of drop-out at Vennesla vgs.

In this article, we illustrate how an explicit approach to time and temporality can provide insight into the development processes of youth employment support. We choose the term ‘illustrate’ since “writing about action research is not the same as doing action research” (Bradbury et al. 2019, pp. 3–4). Writing about action research is not just telling but showing how changes were actually made through action research. The focus of our write-up will be to investigate *how temporality, with the orientation of past, present and future, can be used when working with a complex territorial challenge.* Our investigation corresponds to what the pragmatist philosophers Pierce and Dewey called an inquiry process, a doubtful situation that the inquiry must transform into a problem (Lorino and Mourey 2013). An inquiry does not start with a problem-solving procedure but is a “quest of meaning as a key for action rather than a quest for truth as an accurate copy of reality” (Lorino and Mourey 2013, p. 51).

## Theoretical Discussion

We continue our discussion of temporality as it relates to territorial knowledge co-generation and action research in territorial development (Karlsen and Larrea 2014). In the realist approach, time is conceptualized as chronological and unidirectional, where passage of time is a linear progression from the past to the present and into the expected future (Hussenot et al. 2020). It emphasizes schedules, deadlines, punctuality, synchronization of labour, coordination of activities, and speed. Time is often illustrated in a process with phases or stages along a linear timeline, with a focus on what happened in the past. In this approach, the past has been, and cannot be changed, a desired future can be reached through a planned sequence of interventions organised in a participative manner in the present. Finished past occurrences can be organized along a timeline as stages or phases which can be used to make comparisons or establish cause and effect relationships between different periods of the past and the present. The focus on causality and variables in a variance models means that that there is little room for acknowledging the flow of time, and temporality is either ignored (Cloutier and Langley 2020) or utilized for comparative statistics, before and after evaluation (Pettigrew et al. 2001).

### Process Ontology

The constructivist and process-orientated approach represents an alternative to the realist approach with the ontological assumption that “the world exist as flows in which entities are in a state of becoming rather than as a final state of being” (Hernes 2007, p. 128). By the word ‘flow’, Hernes (2014) means the flow of time where actors organize the world around them in the flow. The openness of the process approach shifts the emphasis from the result of the process to focus on the evolution of the process itself (Langley et al. 2013). The world (reality) is always in a process of changing to something different and is therefore unpredictable. “Process thinking invites reflection on the relationship between the given state of affairs and the multiple possibilities for things to turn out otherwise” (Hernes 2014, p. 3). A linear, clock time orientation is meant to enhance efficiency, coordination, and control while process time is defined as open, organic and cyclical, and may be better suited to “managing emergent, complex and indeterminate processes such as development” (Reinecke and Ansari 2015, p. 618). A process approach does not exclude clock time. There is an increasing recognition that process and clock time can coexist (Reinecke and Ansari 2015). The flows are the organization of ongoing relations or relational processes. According to Pulk (2016, p. 39) examples are: descriptions of routines as flows (Feldman 2000), a flow of possibilities and opportunity driven-choices (Tsoukas and Chia 2002), sensemaking as a flow (Weick 1995) and descriptions of experiences as flow (Bakken et al. 2013). One can interpret an action research process, such as knowledge co-generation process as a flow of actors and their relationships to another.

### Temporality Explained

Temporality, becoming, emergence and perishing are closely related process concepts, as they all refer to change in some shape or form. The ongoing relationship between the past, the present and the future is temporality (Hernes 2014). ‘Ongoing’ signifies what is currently happening, and which actors are in the process of doing (Hernes and Schultz 2013, p. 1). The past, present and future are constantly redefined and reconfigured in the process by the actors and they are therefore not given or stable, but dynamic (Hussenot et al. 2020). The past and the future are not isolated from the present, but partly constitute it. The past is not possible to change since it has happened. “The past is not there ‘in it-self’ (Mead 1932, p. 9) but is called forth in memory through its relationship to the emergent future” (Hernes and Schultz 2013, p. 1). Temporality is acted upon by territorial actors in the knowledge co-generation process. Actors interpret both the past and the emergent future and the narrative about what happened can be revised in any present (Hernes 2014). This implies that what happened in the past can be questioned in the present. The same can apply to the emerging future. Even if the future has not happened, it can be interpreted and imagined differently by the multiple actors in the process. The understanding of the past and the future can therefore be reinterpreted in the present moment. What is important in the co-generative processes is how the iterations of interpretations are utilized to create future actions in the next co-generative event. Interpretations concerning the past event constitutes a knowledge perspective that can be used in the construction of the future.

For territorial actors at the collective level, the explicit awareness is that the present is always providing an alternative view of itself, as part of the ongoing knowledge co-generation process. A different understanding of the past can shift the shared meaning of the present and give form to an alternative view of the present and shape an emergent action for the future that differs from the previous understanding. In flow, the iterations of perspective change can occur consciously and continuously.

An explicit awareness of the present and the development of understanding at any present moment in the knowledge co-creation process provides an alternative view of the past, present and future of a process. Actors engaged in an ongoing process, will experience a flow from the present understanding to a new understanding in the present tense. The continuity of actions that emerges from a co-generation process is thus always in a state of becoming and perishing. Evolution in a co-generation process is a social construction process emerging in the present but pertaining to the future.

### Co-generative Event Explained

In the agora of territorial development, ‘event’ is the term we use to distinguish between the temporal orientation of the past-present-future. Events happen and are experienced in the ongoing present, where evolving knowledge from the past and the future are brought together in the ongoing present (Hernes 2014). ‘Event’ can refer to that which is taking place (*eventum* from Latin) and that which has happened (*eventus* from Latin) (Deroy 2009 in Hussenot et al. 2020). An event is a tangible activity in a moment (Hussenot and Missonier 2015). In the present, an event is the actual moment that actionable knowledge is taking place and happening. An event can last a short time or a long time and can be divided into other events (Hussenot and Missonier 2015). An event can start with decision making, then planning and executing the event, conclude with debriefing and a summary of the results from the event. Each of these moments are in themselves events, but they are events in a process that leads towards a ‘co-generative event’, which is an orchestrated moment in a co-generation process, where invited actors are meeting each other with the purpose of creating new actionable knowledge. A dialogue conference (Gustavsen 1992) or a search conference (Greenwood and Levin 2007) are examples of co-generative events. Events are not discrete entities of finished data or points on a time schedule but are both inputs and outputs of the actual event, they are intertwined and related to each other (Hussenot and Missonier 2015). Each event is linked with the events before and after. Simply put, a co-generation process consists of many co-generative events. In each event, both the past-time and future-time are subject to interpretation by the participating actors. The resulting actions from co-generative events can lead to unexpected developments (McNiff, 2013) which call for further iterations of knowledge co-generation. These unexpected developments can include: the introduction of new elements through increased insight, diversity of perspectives, an alteration of focus or an expansion of scope. Process thinking invites reflection on the relationships between the given state of affairs and the multiple possibilities for things to turn out otherwise (Hernes 2014, p. 3). It is the actors in the process that make the decision of when to stop the process.

In summary, a linear approach to time regards that events, activities, or tasks can be organized along a timeline as entities. In process approach, time is residing within the event, activity, or task, amenable to changing as part of ongoing processes and becoming (Reinecke and Ansari 2015). The past and the future are connected in the present by the actors participating in a co-generative event. A co-generation process consists of many co-generative events where the past and the future is interlinked in each event through the actors participating in the event. See also Table 1 for comparison between linear and process time.

**Table 1:** Linear Time and Process Time

	Linear time	Process time
View of time	“Newtonian” view of time as absolute, unitary, invariant, linear, and mechanical	View of time as subjective, open, relative, organic, and cyclical
Description	Quantitative	Qualitative
Reference frame	Clock based and absolute. Time is an objective measure independent of human experience.	Event based and relative. Time is dependent on human experience. Temporality is integral to the experience of being human.
Orientation	Deadline oriented	Process oriented
Link between past, present and future	Discrete. Past, present, and future as endless “succession of now-points” (Joas 1997).	Continuous. Temporal continuity and ongoing flow of past, present, and future
Logic	Efficiency: Time as scarce resource	Flexibility: Time as contextual feature

Source: Adapted from Reinecke and Ansari (2015, p. 621).

## Fieldwork Discussion

In this section, we describe Project Venneslabrua to represent a point-in-time perspective of our fieldwork that constitutes a retrospective (past event) of our territorial complexity study. Take note of the temporal knowing and time-specific knowledge of the territorial actors involved through time. In the beginning of the 1990s’ a report documented that the Agder region scored low on both living standards and social challenges indicators compared with other regions in Norway (Røed 1993). Agder region is still scoring badly using these same indicators, but lately (since 2018/2019) there has been a shift from focusing on explanation for *why* the region is scoring so low to *how* the situation can be changed.^2^ ‘How to change’ demarcates the start of our fieldwork.

### Collaboration

In our first meeting with the representatives from Agder County about Project Venneslabrua, they said:

*We are interested in collaboration and a co-creation process with the University of Agder in this project (Quoted from our research diary, 22.10.2020).*

The University of Agder had been earlier engaged in Project Venneslabrua, but through faculty researchers of another department. The reason for Agder County’s interest to collaborate with us (Department of Working life and Innovation) was due to our ongoing knowledge and experience with knowledge co-generation processes. In 2020, we collaborated with Agder County in a co-generation project that involved university students and practitioners from the county on the topic of ‘living conditions in Agder’. In our presentation to introduce and review Project Venneslabrua, the first slide was a pictorial of the plan for Venneslabrua with official publication date of 28.10.2018. Two years had already passed since the project plan was published. What had happened in the meantime?

### Challenges

Through a candid presentation of status quo, it was clear that challenges existed regarding project objectives and structure, project leadership, organizational integration of resources, as well as the need for clarification of roles and resolving of tensions and conflict. The responsibility of the project leadership had newly shifted from Vennesla Vgs (the college) to the County Administration in Agder, and it had already been decided that the original project description had to be redrafted. The new project leadership saw a need for revision of the defined goals and structure, and openly stated that tensions and conflicts existed between the current educational professionals and the youth workers who are responsible for the students with social challenges. Among the shared perspectives: youth workers had concentrated their work at an individual level, attempting to assist and support those assigned to them without sufficient integration with the school system. There were other uncertainties about method guidelines for working with the youths, and the various roles and responsibilities within the project. The complexity of the project was apparent.

### Participants

With this information about the past, we started to design the first co-generative event and convened the Advisory Resource Group (ARG) as the main arena for co-generative dialogues. The participating actors for the first ARG comprised of nine participants: two representatives from Agder County Administration, an operational leader of the youth works from Project Venneslabrua, an external resource from a similar project (Project Lindesneslosen) as well as three researchers, an advisor, and a research assistant from the University of Agder. The number of participating actors in ARG varies over time due to the specific purpose of each co-generative event. In events concerned with planning of the seminar and other discussions, it has been important to include participants who can contribute from a variety of perspectives and knowledgebase. Event participation has been adjusted according to the subject matter expertise needed, while core participants remain as the county officials and authors of this article. In general, the number of participants has expanded as the project has developed.

### Planning

**Fig. 1:**
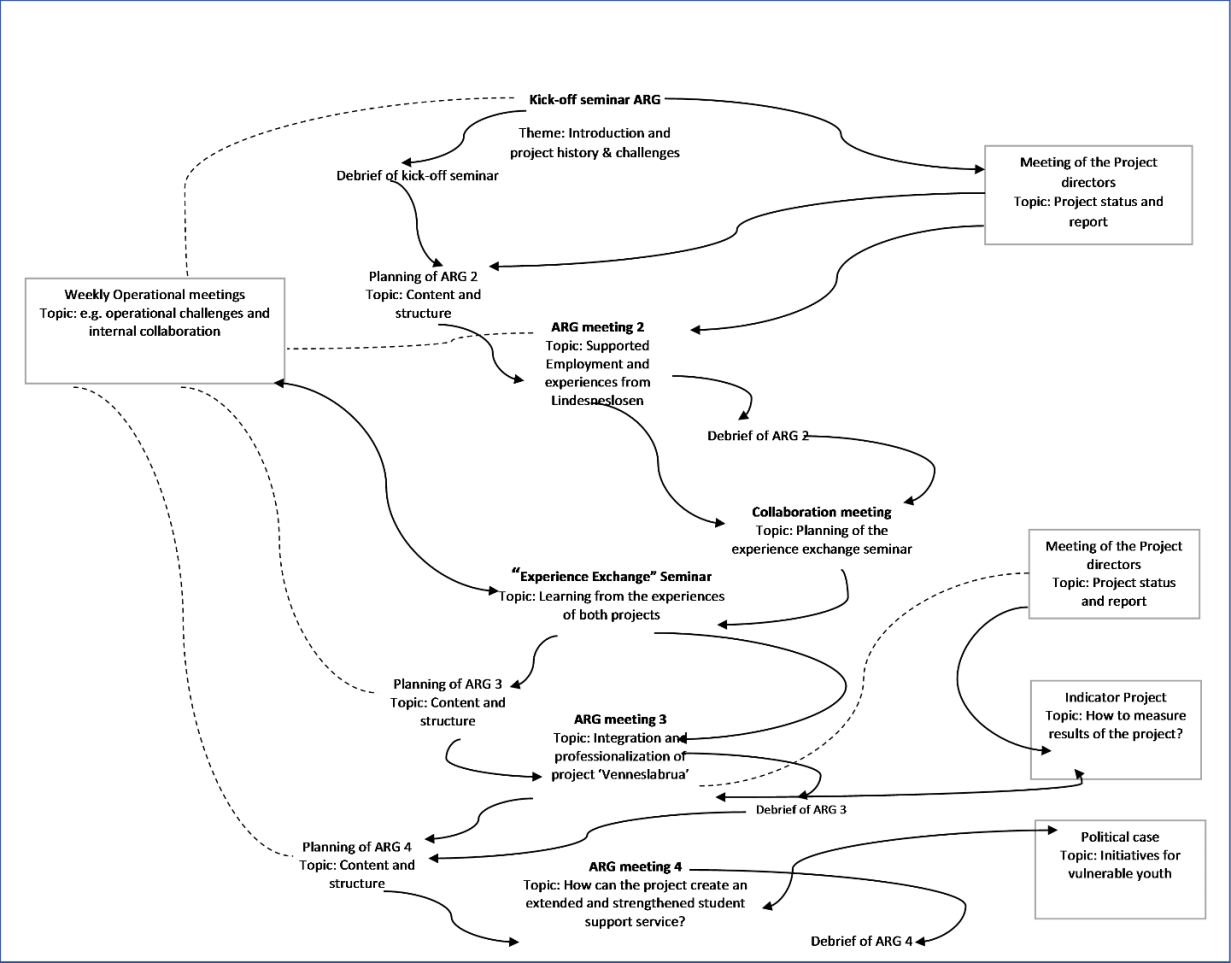
Co-generative Process Flow for Project Venneslabrua

Over a one-year period starting from late 2020 to the end of 2021, four (4) co-generative events have been hosted with the ARG, seven (7) co-generative events and one (1) co-generation seminar have been held within the planning and collaboration group at University of Agder with Agder County officials. Figure 1. Co-generative process diagram, depicts a flow of multiple co-generation events from which perspectives were gathered for this territorial complexity study. The dipiction of the process in Fig. 1 attempts to incorporate meetings and other occurrences that affect the process. Elements are connected using two different line types, whole lines and arrows illustrate a direct connection between elements, both concerning progression of time and the participation of the project group. That is to say, it is simple to follow the line of development of the process in time and participation of the members of the project group using the lines and arrows. The dotted lines connect elements that occurred on the outskirts of the process, that is to say, meetings which occurred, and decisions made parallel to the process timeline and without direct participation by the project members, whilst still having an effect on the process.

We exclude data from other events that have occurred as organizational and systemic activities but include data that directly contribute to the knowledge co-generation process, specifically the regular debriefings with actors from Agder County. In (any process and particularly those of co-generative nature), attention to the culminative flow of events is central (Cloutier and Langley 2020; Langley and Tsoukas 2017), as well as noting the pattern within the flow of events (Hernes 2014). We notice from the process diagram, a flow pattern of various collaborative spaces between researchers and actors: namely a smaller delegation of two (2) county administrators and two (2) researchers known as the Plan & Task Committee that meets to prepare ARG sessions and debriefs.

### Facilitation

Referring to Fig. 1, we draw reader’s attention to the topics discussed in each of the co-generative events. The co-generative events with the ARG were carefully designed by the planning and collaboration group. The content and structure of each discussion were organized in a manner that connected the past and present with the future of the session topic. Every event started with a recap of what had happened since the last time in the project and then an introduction to the topic for the current event. While the tone of dialogue in the events was congenial and constructive, we attempted to scratch the surface of congeniality by asking questions to provoke the exposure of implicit conflict between participants (Karlsen & Larrea, 2016).

### Data collection

Throughout the study period (2020–2021), each ARG meeting attended by researchers was meticulously summarized in the form of minutes, as well as the observations and reflections noted in the research diary of each researcher involved. In the analysis of each co-generative event, the aim is to identify how the actors used and mobilized the past and desired future in their dialogues. We are seeking both the expected as well as the unexpected developments, the iterations knowledge co-generation that may have occurred because of the introduction of new elements through increased insight, diversity of perspectives, the alteration of focus or an expansion of scope.

## Dialogue Process Discussion

Applying awareness of temporality (temporal knowing) in our territorial complexity study, we present a flow narrative of the co-generative events in Project Venneslabrua, as contrasted with a linear description in the fieldwork discussion. Before we start, we want to report that the work with the youths is going well so far. This is an ongoing concern for the Advisory Resource Group (ARG). Since each youth has his/her specific challenges, the youth workers work from an individual approach, not group approach. Each youth worker has the responsibility for following up with 12–15 youths. However, it is too early to say much about the long-term effects of Project Venneslabrua on the student drop-out rate. In the first co-generative event with the ARG, we presented action research for territorial development (Karlsen and Larrea 2014) with an emphasis on territorial complexity and dialogue as the main tool for development inspired by the work of Gustavsen (1992). The following spotlighted dialogues from the selective co-generative events illuminate several challenges: ranging from practicalities that could be easily solved to tensions and conflicts that are escalating in Project Venneslabrua. It is the latter that is the focus in this article.

### Past-time Knowledge

It is impossible to show the entirety of co-generative dialogues, but as an illustration, we quote from the official minutes of one co-generative event with the headline: *project experiences from the first year* dated 17.11.20:

*Ownership and integration in Vennesla Vgs is unclear and must be clarified. Conflict with the existing school advisory service concerning division of responsibilities. (Operational leader of the youth works from Project Venneslabrua*

In our research diary, containing more detailed notes than the minutes from the referenced event, we recorded a citation from an ARG member. She represented another project at Lindesneslosen, that was recently completed at another college in Agder, with almost the same aim as Project Venneslabrua. In the meeting, this ARG member shared:

*… this is like our experience. We were also organized outside the formal organization and located in the inner end of a corridor, away from the rest of the organization we were supposed to collaborate with.*

The reaction of this ARG member was a response to the story told from the coordinator for youth workers in Project Venneslabrua concerning their physical location in a distant corner of the school building. We confess that, in the actual event, we did not reflect more about the connection between these statements, but cognitively the sentences have been living in our heads as something important for the understanding of the process, but without knowing how to connect them. It was not until a later co-generative event that we recognized the connection between these statements from the different events. We will return to this later in the article.

The second co-generative event of the ARG focused on gaining a deeper understanding of the method that had been used in Lindesneslosen.^3^ This session served to reinforce our awareness of the untapped knowledge available from the experiences of Lindesneslosen, which in turn led to the planning and execution of ‘Experience Exchange’ seminar with representatives from Venneslabrua and Lindesneslosen. The aim of the seminar was centered on exchange of knowledge and experience between youth workers in the two projects. The culmination of experiential insights provided the foundation for future co-generative events, where the narrative for Project Venneslabrua shifted toward integration of the project in Vennesla Vgs and learning from Lindesneslosen for the method that Agder Country Administration was using.

### Present-time Knowledge

In the prelude to the ARG’s third co-generative event, operational leader of the youth works from Project Venneslabrua, began the event by commenting *I have a lot on my heart today.* In our research diary we noted: *What does she mean?* Later in the same event we got an answer from her:

*As a representative for the youth workers, I am asking: Are we meant as a reinforcement of student services? Are we supposed to be a part of the existing student resource services? We have established a strong bond with the students, but our work is not recognized/appreciated*

Her direct, engaged voice initiated a long, intense and positive dialogue in the event about possible solutions to the challenge she raised. In an event in the Plan & Task Committee following the third co-generative event with ARG, the project leader from Agder County said that the enthusiasm and energy now was high in Venneslabrua. College advisors had finally started the process of including youth workers from the supportive employment project in their efforts.

In the debriefing after the third co-generative event, we (the authors of the article) started to discuss what had happened in that session. We realised that the operational leader of the youth works from Project Venneslabrua had connected the co-generative dialogue back to the first co-generative event. They (the youth workers) felt like ‘outsiders’ of the formal organization and their claims about this were not taken seriously. This was the same experience as the representative of Lindesneslosen said in the first ARG event. Integration of Project Venneslabrua into the school system in Vennesla vgs. calmed the tensions. This progression carried over into the ARG’s fourth co-generative event, where the topic moved towards the ultimate aim of the project: developing a model for student support that can be applied across the region’s colleges. Given the earlier conflict and struggles in the project, this subject had been kept at a distance to avoid conflict or escalation of tension.

### Future-time Knowledge

From the dialogue in the third co-generative event, we can observe that: on the one hand, it reconstructed the past, and on the other hand changed the future of Project Venneslabrua. The co-generative process diagram (Fig. 1) shows the emergent flow of topics that were discussed in the series of co-generative events that were contributing to redefining the future of Project Venneslabrua. It must be added that the work on redrafting the project plan, which is the official project narrative, has been continuously progressing in the background, carried out by the Agder County officials assigned the role of project leadership team. A co-generated ‘picture’ of Project Venneslabrua is gradually emerging in the present, as envisioned by the active engagement of the participating actors in the co-generative process. However, at present (the actual moment of writing this article) it is still not possible to see what the full picture that is emerging. New changes and challenges can and will appear in the future as unexpected developments.

## Discussion

Through the lens of co-generative events, we bring forward the temporal dimensions of co-generative dialogues that impact the unfolding of Project Venneslabrua. We now speak to how the iterative approach to knowledge co-generation that better address territorial complexity. We cite three escalating tensions that could have evolved into project level conflicts but were identified as pivots of ‘actionable knowledge’. In the narrative we have tried to illustrate how an explicit approach to time and temporality can illustrate and enrich a co-generation process by constructing a process diagram, cf. Figure 1, and a narrative. We will now discuss the process theoretical contributions of the case.

### Conflict between linear time and process time

First, conflict between divergent views of temporality is a new type of conflict that has not been addressed before within action research for territorial development. It is an example of a conflict between linear and process time. When this conflict was presented in the first Advisory Resource Group (ARG) event, we did not, in that moment, acknowledge it as a conflict between different approaches to temporality. Maybe because the linear approach is taken for granted and has become tacit for us, but still implicitly guides our reflection and action. More precisely we are talking about the linearity of territorial development projects, such as Venneslabrua and Lindesneslosen. The basic idea in such projects is that in a specific period of time (2019–2024), extra resources (youth workers) are prioritized to work with a specific purpose (prevent school drop-out) and a specific target group (youths between the ages of 12–15). The assumption is that when a project is finished, the knowledge generated in the project can be transferred like a product, an entity, to other organizations and implemented. Establishing a project implies decoupling it in time from the process it is assumed to influence, i.e., to freeze time and assume that the present is not changed in the ordinary organization and that lessons learned in the future in a project, can easily be transferred back, and implemented. The implicit temporality assumption is that time as a process does not matter, i.e., that the knowledge co-generation process does not matter, it is the outcome that matters. Time is just used as an external measurement instrument for a project, often illustrated with an arrow. This means that time as a process is abstracted away in the project, in the sponsoring organization and in all other organizations the project is meant to be implemented in. The abstraction of time is not a valid assumption, at least for complex processes. As we have shown, actors learn from past experiences and use them in the present to create a desired future. Working with complexity means that the actors in the organizations that are meant to work with the project issue in the future, must and should be involved in the process from the start.

As described from the co-generative events, the participating actors from the collaborating organizations were not engaged from the beginning. Both Project Venneslabrua and Project Lindesneslosen were [located away from and outside] of the formal school system assumed to be their project collaborators. Their outsider status meant that they were neither part of the formal organizational structure, nor integrated into any informal social routines. This [physical separation] assumption and the organizational consequences created conflict in Project Venneslabrua with the established organization, i.e., with Vennesla vgs. This conflict was recognized by an actor in the first ARG’s co-generative event with previous experience from Project Lindesneslosen. The solution after the event was that the project leader from Agder County established a routine of having Wednesday meetings with the actors from Venneslabrua and Vennesla Vgs in order to handle the conflict. These regular meets served to stabilize but not resolve the conflict as the ‘outsider phenomenon’ continues to be present as expressed by the coordinator of youth workers in the third Advisory Resource Group. Since the same pattern showed up in Project Venneslabrua as in Project Lindesneslosen, demonstrates that the temporality tensions were implicit from the moment the projects were established. Project Venneslabrua has now started the process of engaging with the school system and integrating the project idea and method in the ordinary school system. Since this process is just started it is too early to report about the integration.

### Making Sense of the Messiness of Temporality

Utilizing co-generative events and the distinction between the temporal dimensions: past, present and future, enables us to understand how past experiences can lead to change in the present knowing (of actionable knowledge) and the construction of the future even with explicit conflicts. We have used linear time to show the events, but this does not mean that we see each co-generative event as a finished event, that influenced the next event, as in a cause-and-effect relationship. What we have tried to illustrate is that the past was not finished but was brought into the present by the actors and their actions, as happened in co-generative event three. If they were finished the actors would probably not used energy to bring them into the events. [If the outsider phenomenon was resolved from the Wednesday meets, then the questioning of belonging would not have surfaced from the coordinator of youth workers.] Also, the continuous rewriting of the project plan by the project leader shows that the future of the project was changing as the past experiences were reinterpreted by the actors. This demonstrates why one must [maintain a critical perspective] be critical concerning retrospective narratives that tell a story of a knowledge co-generation process following a linear time in phases. The past influences the present and the future in ways that we are not even aware of. “Process data are messy. Making sense of them is a constant challenge” (Langley 1999, p. 691), and any attempt to simplify them is challenging. Tsoukas (2017) argues that researchers should not simplify, as theoretical complexity is needed to account for organizational complexity. Research knowledge and policy decisions ordered along a chronological timeline as different pasts can be subject to interpretation and change in the current present. [Past] Distant knowledge and decisions can therefore be connected and reinterpreted in the present, which can influence the future as [occurred] happened in the first and the third ARG co-generative events.

The continuity of actions that emerge from a co-generation process is always in a state of becoming. Becoming [in the context of territorial development] is understood as the unexpected developments that create the foundation for the construction of the future. Unexpected outcomes (McNiff, 2013) of events can create multiple paths of development [and] of multiple possibilities for progression of the [a co-generative] process. One [unexpected outcome] example is that the conflicts could have [prevented the project from starting] stopped the project before we were engaged in it. Also, after the first co-generative event the project could have been stopped or changed. As we [observed] in the co-generative events, information conveyed in the first ARG meeting led to the uncovering of challenges regarding the early stages of Project Venneslabrua as a project and the use of past knowledge from Project Lindesneslosen. The emergence of this knowledge created the next [actionable knowledge] step in the process. This is an illustration of how the present is connected with the past, as well as inherently interwoven with the future (Hernes 2014; Tsoukas and Chia 2002; Wenzel et al. 2020). In the present [time] tense actors constantly and simultaneously enact and reinterpret the past and the future (Hernes 2014), the culmination of [actionable] knowledge and insights [build] the foundation for a [each] new co-generative event.

### Seeing Time in Movement and with Insider Perspective

Finally, viewing the dynamic nature of [evolution] evolvement from the inside (Shotter 2006), present in the becoming of a process means there is a unique possibility for reflection and iteration. The challenge is how to construct a narrative of [complex processes] where the past, the present and the future are in constant [motion] change. A process narrative can be thought of as a flow of events that when combined make up the whole process. Cloutier and Langley (2020) describe these individual events in a [scripted] narrative as frames in a movie. A film is an example of a temporal flow where the scenes in the movie are events that bring action and continuity in the movie, and for framing a narrative where events and activities are placed in a trajectory that show the evolution of a phenomenon over time (Langley and Tsoukas 2017). Watching the film shows each co-generative event and the co-generative dialogue within leads to changed perspective in actionable knowledge, which in turn leads to changed action. Conveying such a narrative requires consideration of temporal dimensions.

The contribution of modelling the development of the process in the form of the film metaphor is found in the shift in emphasis from the result of the process to focus on the evolution of the process itself (Langley et al. 2013), providing an alternative to the traditional stage or phase perspective on process. In the case utilized in this paper, where the complexity of the context and enormity of the aims rendered the original project plan invalid, it was interesting to follow the development of the process in the present tense, seeing that the evolution of understanding in each co-generative event has created the foundation for progress. For those engaged in this process from the beginning to the present, the results in the project are tangible, from a floundering project shrouded in frustration and uncertainty, the co-generative process has brought the project forward to a functional integration in the existing organization. The narrative from the “Kick-off” to the fourth Advisory Resource Group meeting shows the movement forward, representing results in the form of progress. It can of course be argued that the planning of a process in stages is productive, however we argue that in a complex process such a plan is a mere figurehead of the process.

In modelling the process as we have applied it in this paper allows the representation of parallel activities. In building the narrative of a complex process involving multiple actors, there will be multiple events (Cloutier and Langley 2020) and the process diagram allows us to utilize a narrative to place events and activities in a trajectory, presenting the evolution of a phenomenon over time (Langley and Tsoukas 2017). When focusing on the events and actors in this process-orientated manner, there is a greater danger of developing diagrams so complicated that their meaning is not easily accessible for those outside the project. In this case the events for reflection and actions by actors are defined as co-generative events. The culmination of co-generative events creates the narrative of the co-generative process as a whole. The trajectory shows that there are multiple activities occurring in parallel, for example the need for redrafting of the project plan was clear from the first Advisory Resource Group meeting and work on this can be seen to be undertaken simultaneously. The intention is to avoid the oversimplification of variance models and achieve a representation of complexity, while providing a meaningful diagram.

### Perspective Shifts in Actionable Knowledge by Researchers

As researchers engaged in an ongoing process, we experienced a flow from the present understanding to a new understanding in the present tense. In a retrospective study, the various possibilities would possibly not be represented, if it is not considered primary to describing the ‘end result’, such ambiguity may be considered a weakness in the process. When reviewing a process retrospectively, it is common to reconstruct the story omitting the messy parts, when we know where the process ends it is easier to present a tidy picture of as smooth progression. However, in studying a co-generative process, insights into the multiplicity of developmental paths and construction of the future through knowledge co-generation is considered an enrichment. Note a retrospective study will not necessarily report the same as we have presented, since our process will be finished, and such a study will have another purpose and other questions than the approach we have used. There can therefore be different interpretations of a project as Venneslabrua, depending on research questions and epistemological and methodological approach.

## Conclusion

Our inquiry was to investigate *how temporality, with the orientation of past, present and future, can be used when working with a complex territorial challenge.* The main purpose of this article has been to address time and temporality in co-generated events. Through Project Venneslabrua, we illustrate though the series of four co-generative events how knowledge constructed from the past can be changed in the iterations of knowledge co-generation process. We have used the distinction between past, present now and future to show that though co-generative events the knowledge constructed in the past can be changed. This implies that the past is not finished and that the understanding of the past can be changed through an open dialogue where conflict is made explicit.

In the following section we clarify our contribution to the action research literature concerning co-generation of knowledge. Earlier models for co-generation of knowledge emphasize the importance of various knowledge forms actively participating in the search for collective knowing and new knowledge (Karlsen and Larrea 2014; Elden and Levin 1991; Greenwood and Levin 2007), but did not explicitly represent the role of temporality. Utilizing an event-orientated approach allows for the inclusion of temporality in an explicit manner, emphasizing the advantageous position of the action researcher. An explicit awareness of temporality provides the opportunity for research on evolvement of process from the inside, present in the becoming of a process means there is a unique possibility for reflection and iteration. The proposal is that all participants engaged in an ongoing process will experience a flow from the present understanding to a new understanding in the present tense. An alternative to phase-based process design, modelling the process creates a narrative which tells the story of evolution of the process over time. This is an alternative to retrospective process evaluation, research in the present tense allows for insight into unexpected developments that create the foundation for future action.

### Contribution

To summarize regarding the contributions of this focus on temporal dimensions when researching a process, it is proposed there are three elements that are especially interesting. First an explicit awareness of temporality provides the opportunity for research on evolvement of process from the inside. Present in the becoming of a process means there is a unique possibility for reflection, iteration, and identification of time-related conflicts to first evidence their existence. Second, an alternative to phase-based process design, modelling the process creates a narrative which tells the story of evolution of the process over time, and show actions that followed from the co-generated knowing (happening inside the co-generative events) as the iterations of ‘actionable knowledge’. Shifting emphasis from the result of the process to focus on the evolution of the process itself. Finally, an alternative to retrospective process evaluation, research in the present tense allows for insight into unexpected developments that create the foundation for future action. That some actions don’t resolve/solve the presenting issue (meaning we can’t solve the conflict/tension, maybe it’s good to have them) but rather change the direction of the project or specifically approach.

### Future Research

From the process analysis in the case study, there are a few insights that can be considered enlightening in other contexts. Applying the concept of the ‘co-generative event’, as orchestrated moments where invited actors meeting with the purpose of creating new actionable knowledge, can be a useful alternative to the common “phase” of a process. The co-generative event is a building block in the narrative of the process, carrying insight from past co-generative events into the present tense and creating the future from the reflective process in the co-generative event. This cumulative understanding and representation parallel events provide a more realistic model of the process than a retrospective observation. Regarding engagement of actors and events, this study suggests that involving actors gradually and according to relevance and contribution can be appropriate, it was also apparent that the process progression is not necessarily hindered by disconnects between some of the actors, however investigating this further is perhaps the subject of a new paper. Finally, addressing the topic of integration of the project in a pre-existing organization became central in the progression of the process.

### Limitations

The co-generative process described in this paper occurs within a specified and limited time period, stretching over almost 1 year. The project did not commence at the beginning of the period of study and did not end at the termination of study. This time period was deliberately chosen as it allowed for the elucidation of a progressive co-generation of knowledge at a crucial period of change in the project and it will be exciting to follow the process further in the becoming.

## Data Availability

The raw data is available in Norwegian in a non-redacted format. To respect privacy concerns, redactions are necessary depending on what aspects of the source material are interesting to researchers. Given this qualification, the findings of this study are available from the corresponding author upon request.

## Footnotes

1 Translated directly, the project name ‘Venneslabrua’ means the ‘The bridge in Vennesla’. Vennesla is a small town in Agder. The metaphor of the bridge is meant to represent the purpose of the project in assisting the crossing from college to the workforce.

2 We have been reading policy documents from Agder County but none of the central policy documents are explicit about this change. However, in the co-generative events and in discussions with policy makers they are saying that there has been a shift in the discourse from *why* to *how* among politicians. They (politicians don’t want more descriptions of the living conditions in the region, but actions that can change the situation.

3 This and the rest of the co-generative events were digital due to Covid-19.
